# Sustainable Valorization of Grape Pomace in Sheep Through Systemic Health, Metabolic Safety, Milk and Meat Quality

**DOI:** 10.3390/ijms262110578

**Published:** 2025-10-30

**Authors:** Gabriella Guelfi, Piermario Mangili, Francesca Mercati, David Ranucci, Silvia Crotti, Muhammad Tuseef, Gianluca Veneziani, Vicente Francisco Ratto, Camilla Capaccia, Francesco Ciancabilla, Margherita Maranesi, Cecilia Dall’Aglio

**Affiliations:** 1 Department of Veterinary Medicine, University of Perugia, 06126 Perugia, Italy; david.ranucci@unipg.it (D.R.); vicentefrancisco.rattovalderrama@dottorandi.unipg.it (V.F.R.); camilla.capaccia@dottorandi.unipg.it (C.C.); francesco.ciancabilla@studenti.unipg.it (F.C.); margherita.maranesi@unipg.it (M.M.); cecilia.dallaglio@unipg.it (C.D.); 2 Istituto Zooprofilattico Sperimentale Umbria e Marche “Togo Rosati”, 06126 Perugia, Italy; pm.mangili@izsum.it (P.M.); s.crotti@izsum.it (S.C.); 3 Department of Science, University of Basilicata, 85100 Potenza, Italy; muhammad.tuseef@unibas.it; 4 Department of Agricultural, Food and Environmental Sciences, University of Perugia, 06126 Perugia, Italy; gianluca.veneziani@unipg.it

**Keywords:** grape marc, ruminants, systemic health, milk composition, meat quality, polyphenols, sustainability, feed supplement, circular economy

## Abstract

Grape pomace (GP), a by-product of winemaking, is rich in polyphenols and fiber, making it a promising and sustainable feed supplement for ruminants. This study evaluated the safety and productive impact of a 5% GP-supplemented diet (GP5) including non-lactating end-cycle (EC) ewes regularly destined for slaughter and human consumption, and lactating (LAC) ewes, over a 30-day period. Control (CTRL) animals received a standard pellet diet with no GP inclusion. Sampling was performed at four time points (T0, T10, T20, and T30), corresponding to days 0, 10, 20, and 30 of the experimental period. The study assessed clinical status, hematology/biochemistry (T0 and T30), milk composition (T0, T10, T20, and T30), meat quality traits and oxidative stability in EC ewes (T30). Since no significant differences were observed in the CTRL animals, the effects were evaluated within the GP5 group by comparing T0 vs. T30. Meat quality was assessed by comparing EC-GP5 to CTRL at T30. The GP extract showed a high total phenolic content (254.02 ± 20.39 mg GAE/g DW). No clinical or hematological alterations were observed, and most values remained within physiological ranges. Biochemical analysis revealed significant increases in albumin, bilirubin, creatinine, and triglycerides (*p* < 0.05), with significant decreases in plasma urea and glucose (*p* < 0.05). In LAC-GP5 ewes, milk urea and lactose concentrations decreased (*p* < 0.05), while pH increased (*p* < 0.05), with no significant changes in fat or casein content. These findings are consistent with reduced ruminal propionate availability, leading to decreased hepatic gluconeogenesis and lactose synthesis, with secondary effects on nitrogen metabolism and the acid–base profile of milk. In EC-GP5 ewes, meat quality traits were unaffected, and DPPH scavenging activity did not differ from CTRL (*p* > 0.05). GP5 was metabolically safe, induced adaptive changes in milk composition, and had no negative effects on meat quality, supporting the valorization of grape pomace as a sustainable feed resource. This trial was designed as a metabolic safety assessment, representing a preliminary step toward future mechanistic and molecular investigations.

## 1. Introduction

Grape pomace (GP), the main by-product of the winemaking industry, is characterized by a high content of polyphenols, dietary fiber, residual sugars, lipids, and triterpenes [1]. Its valorization in ruminant feeding represents both an ecological and nutritional opportunity, aligning with circular economy principles by reducing agro-industrial waste while providing functional nutrients to livestock [2,3]. In addition to its sustainable dimension, GP has been proposed as a cost-effective supplement capable of modulating ruminal fermentation, enhancing nutrient digestibility, and contributing to the mitigation of methane emissions [4].

Polyphenols contained in GP, particularly flavanols, and proanthocyanidins, exert systemic bioactivities that extend beyond ruminal modulation. In sheep and other ruminants, they have been linked to improved hepatic function, modulation of nitrogen metabolism, and enhanced antioxidant capacity [5]. Dietary polyphenols can also influence milk yield and composition, where milk is increasingly recognized as a sensitive biosensor of metabolic adaptation in response to nutritional interventions [6]. In meat, GP supplementation has been associated with enhanced oxidative stability and improved shelf life, particularly through its impact on lipid peroxidation and color preservation [7].

Recent studies have further suggested that GP-derived polyphenols act as systemic modulators by targeting redox-sensitive transcription factors (e.g., Nrf2), inflammatory pathways, and epigenetic regulators such as microRNAs, thereby linking nutritional input to molecular and metabolic adaptation in ruminants [8,9,10,11]. Moreover, they may exert intergenerational effects by improving colostrum quality and neonatal immune function, supporting a broader role of maternal nutrition in shaping offspring development [11].

Despite these promising findings, evidence in sheep remains fragmented and often limited to specific productive traits, highlighting the need for integrated studies that simultaneously address systemic health, milk composition, and meat quality under controlled supplementation strategies. Previous investigations have often focused on isolated productive or metabolic traits, with scarce integration of systemic, milk, and meat outcomes under a unified supplementation protocol [5,7]. This gap highlights the need for controlled trials that combine systemic and product-quality endpoints to fully understand the potential of GP in small ruminants, such as sheep and goats.

This study aimed to evaluate the effects of supplementing sheep diets with 5% grape pomace (GP5) on systemic health, metabolic safety, milk physicochemical properties, and meat quality, while verifying the maintenance of overall physiological balance. The 5% inclusion rate was selected based on previous studies showing that grape pomace levels around this threshold effectively improve metabolic balance and product quality without impairing feed intake or digestibility in ruminants [7,12,13]. By integrating hematological, biochemical, and product-quality assessments (milk and meat), the study provides a comprehensive evaluation of GP5 as a sustainable dietary supplement. Furthermore, it may lay the groundwork for future research exploring the epigenetic mechanisms that could underlie protein-level adaptations and phenotypic changes in systemic functions and production outcomes.

## 2. Results

### 2.1. Polyphenol Content and Antioxidant Activity of GP Extract

Extraction led to the isolation of a polyphenol-rich dry extract, with an average yield of 5.58 ± 0.10%. Total phenolic content and antioxidant activity are reported in Table 1.

The GP extract demonstrated notable antioxidant activity, supported by a high total phenolic content (254.02 ± 20.39 mg GAE/g DW). Among the assays, the FRAP and DPPH tests yielded particularly strong results (1309.63 ± 64.51 and 1110.97 ± 13.39 mg TE/g DW, respectively). The ABTS assay value was 139.55 ± 2.92 mg TE/g DW. The extract also contained measurable levels of anthocyanins (3.94 ± 0.13 mg/g, expressed as malvidin-3-O-glucoside equivalents). These findings are consistent with previous studies, such as that by Ponticelli et al. [14], which highlighted the effectiveness of hydroalcoholic solvent systems, like the one employed in this work, for obtaining antioxidant-rich extracts from GP. Further characterization by UHPLC–HRMS confirmed the presence of multiple polyphenolic compounds, based on the retention times and MS/MS fragmentation profiles provided in Supplementary Table S1. Twenty-nine compounds were identified, of which 12 were identified and confirmed by comparison with reference standards. The identified compounds were quinic acid (1), malic acid (2), tartaric acid (3), procyanidin B2 (6), catechin (7), procyanidin like (8), epicatechin (10), Procyanidin like (13), quercetin 3-β-d-glucoside (17), naringenin (19), quercetin (20), luteolin (22), maslinic acid (26), and ursolic acid (28) profiles provided in Supplementary Figure S1.

### 2.2. Nutritional Traits of GP-Enriched Diet

To evaluate the nutritional impact of GP inclusion, the analytical composition of the CTRL and GP5 diets was determined. The inclusion of 5% GP in the experimental diet resulted in minor changes in the proximate composition. Compared to the control diet, the GP5 diet showed a modest increase in fiber (CF: 7.02% vs. 5.65%) and lignin content (LG: 3.93% vs. 3.58%), with a slight reduction in crude protein (CP: 15.24% vs. 15.43%). The ASH (as total mineral residue after combustion) content and macromineral concentrations (Ca, P, and Na) remained comparable between the two formulations. Total dry matter content was 86.78% for the control diet and 86.92% for the grape pomace diet. The analytical composition of the control and GP5 diets is reported in Table 2.

### 2.3. Veterinary Surveillance

Animals showed no clinical signs of ruminal dysfunction or systemic illness during the experimental period. No cases of acidosis-related manifestations (decreased feed intake, altered rumination, diarrhea, discomfort) were observed. No treatments or veterinary interventions were required.

### 2.4. Systemic and Metabolic Profile

Systemic and metabolic safety were assessed through an extensive hematological and biochemical evaluation. Hematological parameters included white blood cell count (WBC), lymphocytes (LYM), monocytes (MONO), granulocytes (GRAN), hemoglobin (HGB), hematocrit (HCT), red blood cell count (RBC), mean corpuscular volume (MCV), mean corpuscular hemoglobin (MCH), mean corpuscular hemoglobin concentration (MCHC), red cell distribution width (RDW and RDWa), platelet count (PLT), and mean platelet volume (MPV). The biochemical profile comprised hepatic and biliary enzymes (albumin, ALB; alkaline phosphatase, ALP; alanine aminotransferase, ALT; aspartate aminotransferase, AST; gamma-glutamyltransferase, GGT; bilirubin, BIL), renal function markers (urea, UREA; creatinine, CREA), metabolic and muscle indicators (creatine phosphokinase, CPK; cholesterol, CHOL; triglycerides, TG; total proteins, TP; ketone bodies, KET; glucose, GLU; lactate dehydrogenase, LDH), and mineral metabolism parameters (calcium, Ca; phosphorus, P).

Systemic Health—In CTRL animals, significant differences were observed neither between T0 and T30 nor when compared with GP5 animals at baseline (T0). Therefore, treatment-related effects were specifically evaluated by comparing GP5 animals at T0 and T30.

Most parameters remained within or close to physiological ranges; mild deviations (e.g., PLT slightly below the lower reference limit and RDW slightly above) were clinically unremarkable. To maintain consistency, hematological data from EC and LAC animals were evaluated together and are presented as combined mean values (Table 3). Platelet count showed a small, non-significant fluctuation (T0: 188 ± 45 vs. T30: 191 ± 57 × 10^9^/L; *p* > 0.05), consistent with biological and method-related variability (impedance counting and occasional EDTA-related platelet clumping). Values remained close to laboratory-specific reference intervals and had no clinical relevance (Table 3).

Metabolic safety—In CTRL animals, significant differences (*p* > 0.05) were observed neither between T0 and T30 nor when compared with GP5 animals at baseline (T0). Therefore, treatment-related effects were specifically evaluated by comparing GP5 animals at T0 and T30. ALB increased significantly (*p* < 0.05); ALT showed a non-significant increase, and AST a non-significant decrease (*p* > 0.05); BIL increased significantly (*p* < 0.05), while ALP and GGT did not vary significantly (*p* > 0.05). UREA decreased significantly (*p* < 0.05), while CREA increased significantly (*p* < 0.05). TG increased (*p* < 0.05) and glucose decreased (*p* < 0.05), while CPK, LDH, TP, KET, calcium, and phosphorus showed no significant changes (*p* > 0.05). Fat, casein, and protein contents were also unaffected (*p* > 0.05) (Table 4).

### 2.5. Milk Physicochemical Properties

In the CTRL group, significant differences were neither detected in any milk parameter across the experimental time points (T0, T10, T20, and T30), nor between CTRL T0 and GP5 T0 (see Supplementary Table S3). These data confirm that the observed changes in GP5 are not due to natural fluctuations over time or baseline differences between groups, but are specifically associated with the dietary treatment. In GP5 animals, significant differences were observed in several milk composition parameters between T0, T10, T20, and T30. Specifically, lactose concentration decreased significantly (*p* < 0.05) from T0 to T30, with the highest levels at T0 (4.66 ± 0.09%) and the lowest at T30 (0.87 ± 0.42%). Similarly, urea concentration decreased significantly (*p* < 0.05), with T0 at 58.31 ± 2.43 mg/dL and T30 at 17.26 ± 1.91 mg/dL. pH increased significantly from T0 to T30 (*p* < 0.05), with values of 6.80 ± 0.08 at T0 and 7.39 ± 0.05 at T30. Finally, acidity decreased significantly (*p* < 0.05) from T0 to T30, with T0 at 5.40 ± 0.50 and T30 at 3.68 ± 0.17. No significant changes were observed in fat, casein, or total protein content (*p* > 0.05) (Table 5).

All samples tested negative for mastitis-associated bacteria, and SCC values ranged between 120 × 10^3^ and 340 × 10^3^ cells/mL, remaining below the physiological threshold for ovine milk (<500 × 10^3^ cells/mL) [16].

### 2.6. Meat Characteristics and Oxidative Properties

In CTRL animals, no significant differences were observed; therefore, meat quality was specifically evaluated by directly comparing EC-CTRL and EC-GP5 animals at T30. No significant differences were detected between EC-CTRL and EC-GP5 animals at T30 for any physicochemical, texture, or oxidative parameter. Meat oxidative stability, evaluated by TBARs and DPPH assays, was not affected by GP5 supplementation (*p* > 0.05) (Table 6).

## 3. Discussion

Supplementing sheep diets with 5% grape pomace (GP5) resulted in consistent metabolic changes, observed systemically and in milk composition, while preserving clinical stability and hematological integrity. This study was designed as a safety and metabolic trial rather than a mechanistic investigation, providing essential baseline evidence before exploring molecular pathways and gene-level mechanisms in future research. Most parameters remained within or close to physiological ranges, with mild, clinically irrelevant deviations. CTRL animals showed significant changes neither between T0 and T30, nor differences at baseline compared with GP5 animals, confirming group homogeneity and the absence of baseline bias. Therefore, data from GP5 animals were analyzed by combining EC and LAC groups to increase statistical power and reduce inter-group variability. The slightly higher crude fiber content of the GP5 diet reflects the intrinsic composition of grape pomace and was not corrected, as both diets were formulated to be isoenergetic and isonitrogenous within recommended nutritional ranges. From a metabolic perspective, GP5 supplementation induced clear shifts in selected biochemical parameters. The increase in serum albumin indicated improved hepatic protein synthesis and systemic protein status [17]. The compositional analysis of the GP extract revealed high antioxidant capacity (FRAP, DPPH) and a diverse polyphenolic profile, including catechin, epicatechin, quercetin derivatives, and triterpenes such as maslinic and ursolic acids, these values were obtained from our grape pomace extract, which was analyzed following the same hydroalcoholic extraction and spectrophotometric methods described by Ponticelli et al. (2025) [14]. These bioactive compounds, widely recognized for their antioxidative, anti-inflammatory, and metabolic-modulating actions, likely underpinned the observed physiological adaptations. The main polyphenols identified in our extract, catechins, procyanidins, and flavonols (as quercetin), are known to modulate glucose metabolism and redox balance, supporting the observed reduction in blood glucose without repeating numerical data [18]. In ruminants, tannins and proanthocyanidins bind dietary proteins in the rumen and reduce their degradation, leading to lower ammonia production and, downstream, reduced blood and milk urea concentrations, which is consistent with our results [19,20,21]. Moreover, grape pomace supplementation has already been shown to exert favorable effects on milk composition in both small ruminants and dairy cows, further supporting the biological plausibility of our findings [5,22]. ALT exhibited a modest, non-significant rise, whereas AST showed a non-significant decrease. The moderate increase in bilirubin, accompanied by unchanged hepatic enzyme activity and the absence of clinical signs, supports the hepatoprotective and antioxidant effects of grape polyphenols, which are known to enhance detoxification pathways and promote hepatocellular autophagy [10,23]. Bilirubin itself acts as a physiological antioxidant, and polyphenols may amplify this function through Nrf2-mediated activation of phase II detoxification enzymes. These findings support the hepatoprotective and antioxidant effects of grape polyphenols, which are reported to enhance detoxification pathways and promote hepatocellular autophagy [10]. At low-to-moderate concentrations, unconjugated bilirubin is increasingly recognized as a systemic antioxidant associated with reduced oxidative stress, lower cardiovascular risk, and enhanced metabolic resilience. Polyphenols may potentiate these effects by activating Nrf2-mediated transcriptional pathways, thereby promoting phase II detoxification enzymes and cellular defense mechanisms [12]. Nitrogen metabolism displayed contrasting adaptations: GP5 supplementation was associated with a decrease in plasma urea, an increase in creatinine, and a decrease in glucose; triglycerides increased, whereas ALT, AST, TP, LDH, and CPK did not change significantly. Most variations remained within physiological ranges, confirming the safety of GP5 supplementation. Energy metabolism was also affected, indicating greater lipid mobilization and altered carbohydrate utilization consistent with previous reports of polyphenol-mediated improvements in energy homeostasis and insulin sensitivity in ruminants [7,24].

The marked reduction in lactose observed in GP5 animals reflects the decreased availability of blood glucose, the primary precursor for mammary lactose synthesis. In ruminants, starch and sugars are fermented into volatile fatty acids (VFA), among which propionate is the primary ruminal VFA supporting gluconeogenesis. By contrast, acetate, although quantitatively abundant, is not converted into glucose, whereas propionate is absorbed and converted into glucose in the liver [25,26]. Grape pomace, rich in insoluble fiber and polyphenols, reduces the fraction of fermentable carbohydrates and shifts ruminal fermentation toward acetate, thereby limiting the generation of gluconeogenic propionate [26]. In addition, its polyphenolic fraction exerts antimicrobial effects on propionate-producing microorganisms, further influencing milk composition [12]. The resulting reduction in absorbed propionate limits hepatic gluconeogenesis, as confirmed by decreased blood glucose levels at T30, thereby restricting lactose synthesis in milk at T30. This decline was paralleled by a decrease in milk urea, suggesting reduced nitrogen turnover, and by alterations in the milk acid–base profile, with increased pH and decreased titratable acidity. Collectively, these coherent changes indicate that the reduction in lactose and associated parameters is a direct consequence of diminished propionate availability. Prolonged or higher GP inclusion rates might further reduce milk solids, and therefore a 5% supplementation is recommended as an optimal and safe threshold. The observed decrease in milk lactose and urea in GP5-fed ewes reflects a metabolic adaptation rather than a detrimental effect. Polyphenols and tannins contained in grape pomace reduce the ruminal degradability of carbohydrates and proteins, limiting propionate and ammonia production. As a consequence, the lower systemic availability of glucose and urea precursors leads to decreased synthesis of lactose and milk urea nitrogen. This mechanism supports improved nitrogen utilization efficiency and reduced ruminal ammonia losses, consistent with previous studies on polyphenol-supplemented ruminants. However, if maintained over a prolonged milking period or at higher inclusion rates, such changes could reduce milk solids; therefore, a 5% supplementation appears to represent an optimal and safe level for maintaining both metabolic balance and milk quality. Microbiological testing was negative for mastitis-associated pathogens, and SCC remained within physiological thresholds for sheep milk [16], thereby confirming the absence of pathological confounders and validating that the observed changes reflected the nutritional effects of GP5 supplementation. This trial was not designed to monitor milk yield, but rather to specifically evaluate systemic safety and compositional traits. To control for stage-of-lactation effects on milk traits, animals were sampled at a single, well-defined stage (late lactation), as stage of lactation is known to markedly affect milk composition and related phenotypes in Sarda dairy ewes [13]. We chose late lactation rather than early lactation because early lactation is characterized by high metabolic stress, negative energy balance, and greater individual variation among animals [27], whereas late lactation tends to offer more physiological stability with less between-animal variability in metabolic and hematological parameters [28]. These compositional shifts may have implications for the offspring: reduced lactose and urea, together with increased milk pH, could reflect maternal metabolic adjustments with potential benefits for suckling lambs [28]. Consistently, dietary grape pomace supplementation has been shown to modify the phenolic profile of ewe milk, confirming its role as a sensitive biosensor of nutritional input [22]. Moreover, grape pomace and seed supplementation has been reported to affect milk composition in ewes, further supporting the responsiveness of milk to dietary polyphenols [29]. While the present study did not address this dimension directly, the hypothesis that GP-derived nutritional changes could indirectly influence offspring development via such signaling pathways warrants cautious consideration in future research.

Meat quality was unaffected by GP5 supplementation. No significant differences were detected between EC-CTRL and EC-GP5 animals at T30 for physicochemical, textural, or oxidative traits, including color attributes, water-holding capacity, tenderness, and lipid oxidation. Similar results have been reported in previous studies, where grape pomace supplementation in ewe diets did not negatively influence lamb meat quality and was associated with improved in water-holding capacity [30]. TBARs values and Warner–Bratzler shear force measurements confirmed that GP5 supplementation preserved the technological quality of meat, in agreement with Vieira et al. [7] and with previous evidence on the role of dietary polyphenols. The absence of changes in TBARs DPPH values confirms that 5% GP inclusion does not alter meat oxidative stability, consistent with reports describing limited transfer of dietary polyphenols to muscle tissue. Consistently, the DPPH radical scavenging assay showed no significant differences between groups. This finding, together with TBARs values, indicates that GP5 supplementation preserved the oxidative stability of meat. The absence of adverse changes in radical scavenging capacity, together with TBARs and WBSF results, and color attributes, reinforcing the technological safety of GP5 in sheep diets [31]. As a limitation, carcass weight, body condition score, meat yield, proximate composition, initial and final body weight, feed intake, feed conversion ratio, and mean lactation day were not recorded, as the trial was specifically designed to assess systemic safety and product quality traits under a fixed daily ration. In addition, milk yield was not recorded, which represents a further limitation; however, several studies have already investigated the effects of grape pomace on productive performance in sheep. The present trial was instead designed to assess metabolic safety and compositional traits under controlled dietary conditions. Milk yield was not recorded, as this study was specifically designed to evaluate metabolic safety and compositional traits under a fixed daily ration. Future trials will incorporate milk yield assessment to clarify whether grape pomace supplementation affects productive performance. While these zootechnical parameters would provide complementary insights, they will be addressed in future trials focused on productive performance for a more comprehensive assessment of the impact of grape pomace supplementation. Nevertheless, the 30-day duration limits the assessment of whether the observed adaptations represent transient adjustments or more persistent responses. Moreover, the relatively small sample size may have reduced the ability to capture inter-individual variability, reflecting the exploratory design of this metabolic safety trial and underscoring the need for larger studies across breeds and production systems. Future investigations involving larger cohorts should also include direct rumen VFA profiling to validate the propionate-mediated mechanism. As a general consideration, some limitations are associated with the use of grape pomace as a feed ingredient. Its composition may vary widely depending on grape variety, winemaking process, and storage conditions, potentially influencing the nutritional and polyphenolic profile. The high fiber and tannin content, if not properly balanced, could reduce diet palatability and nutrient digestibility. Moreover, the lack of long-term studies and standardized formulations currently limits the extrapolation of results to broader production systems. The present study provides a solid platform for future investigations into the molecular and epigenetic mechanisms underlying diet-induced adaptations. Ongoing next-generation sequencing of the miRNome at T0 and T30 is expected to provide genomic-level insights that will complement the present physiological findings, consistent with the recognized role of polyphenols as modulators of DNA methylation, histone acetylation, and microRNA networks in ruminants [32]. These findings reinforce the rationale for considering GP as a sustainable and functional feed resource in small ruminant production.

## 4. Materials and Methods

### 4.1. Animal Enrollment

The animal study protocol was approved by the Ethics Committee of the University of Perugia (protocol code n. 46269, approval date: 14 February 2023). A total of 22 healthy Sarda dairy ewes, a Mediterranean dairy breed widely used in Central Italy and homogeneous in age and rearing conditions, were enrolled, including lactating ewes in late lactation and non-lactating end-cycle animals destined for slaughter. All animals were adult ewes aged between 3 and 5 years old, with an average body weight of approximately 55 ± 5 kg. Based on their physiological status, animals were classified into two experimental groups: End Cycle (EC), non-lactating sheep destined for slaughter and human consumption, and Lactating (LAC) sheep. End-cycle ewes were non-lactating animals regularly destined for human consumption, and slaughtering was performed in a licensed abattoir in compliance with Council Regulation (EC) No. 1099/2009 on the protection of animals at the time of killing and national legislation (Law No. 333/98; Council Directive 93/119/EC). Although animals were assigned to EC (non-lactating) and LAC groups according to their physiological status, all sheep were homogeneous in terms of breed, age, health status, and rearing conditions.

### 4.2. Experimental Design and Dietary Treatment

**Figure 1:**
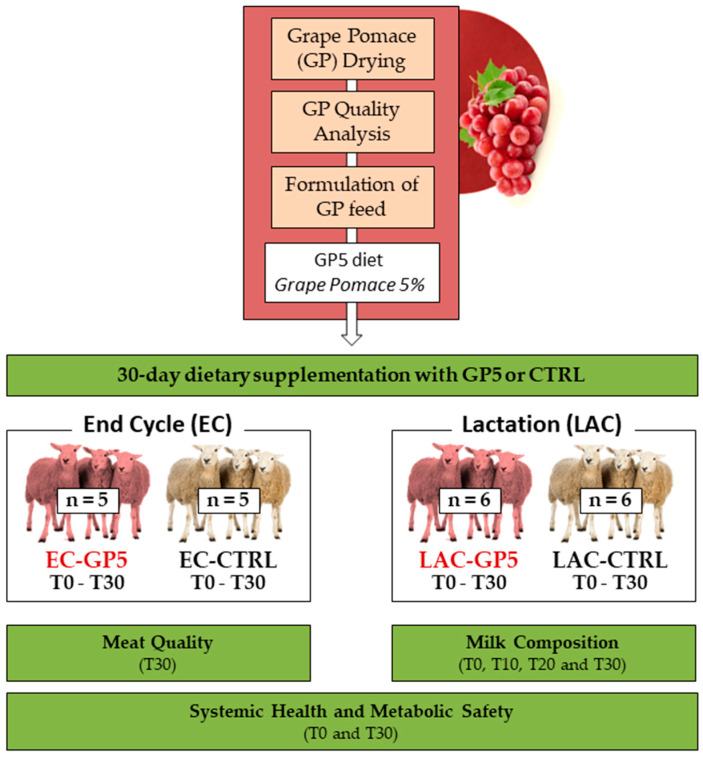
Experimental design. Grape pomace (GP) was dried, analyzed, and incorporated at 5% into the experimental feed (GP5). Thus, the GP5 diet consisted of 95% of the control formulation plus 5% grape pomace. Twenty-two sheep were assigned to two physiological groups: End Cycle (EC, *n* = 10) and Lactating (LAC, *n* = 12), each including animals fed either a control or GP5 diet. Lactating ewes were in late lactation (~5 months postpartum) with an average milk yield of ~0.5 L/day. Animals were monitored from day 0 (T0) to 30 (T30). Systemic health and metabolic safety were assessed in both groups (T0 and T30); meat quality was evaluated in EC animals at slaughter (T30), whereas milk composition was analyzed in the LAC group (T0, T10, T20 and T30).

The experimental design aimed to investigate the effects of dietary supplementation with 5% GP on sheep systemic health, metabolic safety, milk composition, and meat quality. GP was first dried using a fluidized bed dryer and analyzed for its nutritional and functional properties. GP was included at 5% into the control diet to formulate the experimental diet. Importantly, grape pomace was incorporated into the already finished feed pellets, rather than during the industrial pelleting process, thereby preventing any risk of polyphenol degradation. Thus, the GP5 diet consisted of 95% of the control formulation plus 5% grape pomace. The inclusion rate of 5% was selected based on previous literature indicating this level as a safe and effective threshold for small ruminants. All animals were fed individually a fixed daily ration of 800 g/day (CTRL or GP5), administered in two equal portions of 400 g in the morning and evening; individual feed intake and feed conversion ratio were not calculated. Sheep were allocated into two groups: End-Cycle (EC, *n* = 10; non-lactating animals destined for slaughter at T30) and Lactating (LAC, *n* = 12), each including animals fed either a control or GP5 diet. Moreover, the relatively small sample size reflects the exploratory design of this metabolic safety trial, which was intended to identify coherent physiological responses rather than population-level variability. T0, T10, T20, and T30 indicate the sampling days (day 0, 10, 20, and 30, respectively). The experiment followed a completely randomized design with two dietary treatments (CTRL and GP5) and two physiological stages (EC and LAC), each sampled at T0 and T30. At the beginning of the trial, lactating ewes were in late lactation, approximately five months postpartum, with an average daily milk yield of ~0.5 L per animal. Weekly veterinary inspections were performed. Blood samples were collected at T0 and T30 to assess systemic health and metabolic safety. Meat quality was evaluated at T30 in EC animals only, while milk composition was analyzed at T0, T10, T20, and T30 in LAC groups (Figure 1).

### 4.3. GP Drying, Extraction, and Polyphenol Characterization

Wine pomace (400 kg; 55% moisture) from red wine production was dried using a fluidized bed dryer (Costruzioni Apparecchiature Meccaniche—CAM, Cerro Maggiore, MI, Italy). Batches of 30 kg were processed at 70 °C for 1 h, reaching a final moisture content of 8%. The dried material (170 kg) was stored in 25 kg feed-grade bags in a dry environment until use in animal diet formulation. GP (dry weight) was extracted in a 50:50 (*v*/*v*) ethanol–water solution acidified with 1% HCl (1 M), using a solid-to-solvent ratio of 1:5 (*w*/*v*). Extraction was performed in a thermostatically controlled shaking water bath (Memmert, Schwabach, Germany) at 60 °C for 2 h, with continuous forward/reverse agitation maintained at 150 strokes per minute. The resulting mixture was filtered through filter paper with a pore size of 10–20 µm, then centrifuged for 5 min at 4500 rpm at 20 °C. There were three repetitions of this step. A rotary evaporator set to 37 °C was used to concentrate the combined supernatants under lower pressure. Using spectrophotometric tests in 96-well microplates, the dry GP extract’s bioactive profile and antioxidant activity were evaluated. While antioxidant capability was assessed using the DPPH, ABTS, and FRAP assays, with absorbance measured at 515 nm, 734 nm, and 593 nm, respectively, total phenolic content (TPC) was calculated by measuring absorbance at 723 nm. The anthocyanin concentration was determined by measuring absorbance at 538 nm, and the results were expressed as milligrams of malvidin-3-O-glucoside equivalents per gram of extract. All absorbance measurements were made using a UV-Vis spectrophotometer (SPECTROstarNano, BMG Labtech, Ortenberg, Germany). Results were reported as milligrams of gallic acid equivalents (GAE)/g for TPC, milligrams of malvidin-3-O-glucoside equivalents/g for anthocyanins, and milligrams of Trolox equivalents (TE)/g for antioxidant activity. All measurements were performed in triplicate using the procedures outlined by Ponticelli et al. (2025) [14], with the exception of the ABTS assay [33]. Polyphenolic characterization was performed on the grape pomace extract, which represented the functional ingredient added to the diet. The final pellets were not analyzed because the inclusion of grape pomace at only 5% of the formulation diluted the polyphenolic fraction to levels too low for accurate quantification. The stability of the polyphenolic fraction was preserved because grape pomace was added after pelleting, thus avoiding heat-related degradation during feed processing. This represents a limitation of the study, as direct confirmation of polyphenol levels in the final feed matrix was not performed. The GP extract was analyzed using an ultra-high-performance liquid chromatography system (Vanquish UHPLC, Thermo Scientific, Waltham, MA, USA) and a high-resolution mass spectrometer (Orbitrap Exploris™ 120, Thermo Scientific). Chromatographic separation was performed on a Phenomenex PS C18 column (Phenomenex Inc., Torrance, CA, USA) (100 × 2.1 mm, 1.6 µm, 100 Å) at 30 °C. The mobile phases consisted of water with 0.1% formic acid (solvent A) and methanol with 0.1% formic acid (solvent B), applied according to the following gradient: 0–5 min, 5–35% B; 5–25 min, 35–100% B; 25–35 min, 100% B. Flow rate was set at 0.2 mL/min, and the injection volume was 5 µL. Mass detection was performed in negative ionization mode (ESI−), with a capillary voltage of 3500 V, resolution of 120,000 (Full MS and dd-MS^2^), and *m*/*z* acquisition range of 80–1200. Nitrogen was used as the collision gas, with a CID energy of 30 eV. Instrument parameters included an ion transfer tube temperature of 300 °C, vaporizer temperature of 280 °C, and sheath, auxiliary, and sweep gas values of 40, 20, and 0 (arbitrary units), respectively. EASY-IC™ was applied for internal calibration. Compounds identification was based on retention time and fragmentation patterns compared to standard references. Data acquisition and quantification were performed using Chromeleon™ 7.3.1 software (Thermo Scientific Dionex™).

### 4.4. Pelleted Feed Formulation

The CTRL diet was composed of standard raw materials commonly used in ovine nutrition. The GP5 diet maintained a similar CTRL composition with the inclusion of 5% GP. Both diets were formulated to be isoenergetic and isonitrogenous, as verified through proximate chemical analysis (Table 2), which confirmed comparable energy and protein content between CTRL and GP5 formulations [15]. In particular, diets were balanced according to the NRC (National Research Council) 2007 recommendations for small ruminants [15], which represent the standard nutrient levels for high-yielding dairy sheep in intensive production systems. The detailed composition of the mineral–vitamin premix, corresponding to commercial formulations routinely used in intensive ovine nutrition, is reported in Supplementary Table S2. Diets were provided exclusively as pelleted feed composed of the ingredients listed in Table 7.

### 4.5. Systemic Health and Metabolic Safety

Hematological parameters were analyzed in blood samples from EC and LAC animals (CTRL and GP5) at T0 and T30 to evaluate systemic health and metabolic safety.

The health parameters were included to assess immune function, erythrocyte status, and overall health: total and differential white blood cell counts, including total leukocytes (WBC), lymphocytes (LYM), monocytes (MONO), granulocytes (GRAN), erythrocyte indices, including red blood cell count (RBC), hemoglobin concentration (HGB), hematocrit (HCT), mean corpuscular volume (MCV), mean corpuscular hemoglobin (MCH), and mean corpuscular hemoglobin concentration (MCHC); and platelet-related parameters, including platelet count (PLT), mean platelet volume (MPV), and red cell distribution width (RDW, expressed as RDW% and RDWa). In addition, to investigate potential functional effects of dietary supplementation, a panel of biochemical parameters was evaluated at both T0 and T30. These were categorized into functional groups: hepatic enzymes and biliary profile, including alanine aminotransferase (ALT), aspartate aminotransferase (AST), alkaline phosphatase (ALP), gamma-glutamyltransferase (GGT), albumin (ALB), and bilirubin (BIL); renal function markers, including urea (UREA) and creatinine (CREA); muscle enzymes and metabolic indicators, including creatine phosphokinase (CPK), lactate dehydrogenase (LDH), cholesterol (CHOL), triglycerides (TG), total protein (TP), ketone bodies (KET), and glucose (GLU); and mineral metabolism indicators, including calcium (Ca) and phosphorus (P). Changes observed between T0 and T30 within these parameters were used to assess possible metabolic modulations induced by the GP5 diet. Blood was collected into two types of plastic tubes: one containing EDTA for hematological analysis and one without anticoagulant for serum separation. Biochemical evaluations were performed within 6 h of collection. EDTA-treated samples were immediately analyzed for complete blood counts using an Exigo EOS 90 analyzer (Boule Medical AB^®^, Stockholm, Sweden). Tubes without anticoagulant were centrifuged at 2500× *g* for 15 min to obtain serum, which was then stored at −20 °C until analysis. Biochemical analyses were carried out using a Konelab™ 200i chemistry analyzer (Thermo Scientific^®^, Thermo Fisher Scientific Inc., Waltham, MA, USA).

### 4.6. Milk Composition Analysis

Milk samples from LAC animals (CTRL, GP5) were collected at T0, T10, T20, and T30 in sterile vials and transported on ice for immediate analysis. Compositional parameters, including fat, total protein, caseins, lactose, and urea, were quantified by mid-infrared spectroscopy using a Milkoscan™ 7RM (Foss, Hillerød, Denmark). Milk acidity was assessed through a combined determination of pH and titratable acidity (°SH) within the same analytical procedure. This method provides an integrated measure of the acid–base balance of milk, as it reflects not only the concentration of free hydrogen ions but also the buffering capacity arising from several components such as caseins, phosphates, citrates, and lactate.

Microbiological quality was evaluated by plate culture on non-selective blood agar, with 10 µL of each sample inoculated for bacterial detection. Selected colonies were subsequently re-streaked and identified by MALDI-TOF mass spectrometry (Bruker Daltonics, Bremen, Germany). Total mesophilic bacterial count at 30 °C was not performed, as it is regulated by EU law (Reg. 853/2004) on bulk tank milk and reflects milking hygiene, equipment sanitation, and cooling practices, without correlation to individual diet or udder health.

Somatic cell count (SCC) was determined with a Fossomatic™ 7 (Foss, Hillerød, Denmark), and the results were interpreted according to established physiological thresholds for ovine milk, generally under 500,000 cells/mL in healthy animals [16].

### 4.7. Meat Quality Analysis

Animals were regularly slaughtered in a local slaughterhouse, and after bleeding, skinning, and evisceration, carcasses were promptly refrigerated at 5 ± 1 °C. *Longissimus dorsi* muscle samples (10 × 10 cm) were obtained at the last thorax vertebrae after 24 h of chilling from both right and left sides. Samples were stored in sterile plastic bags and transported under refrigerated conditions to the laboratory for analytical determinations. Muscle pH was measured using a pH meter (HI 5221, Hanna Instruments, Woonsocket, RI, USA) equipped with a penetration electrode. At 24 h post-mortem, the following meat quality traits were evaluated: color, drip loss, cooking loss, and Warner–Bratzler shear force (WBSF) [34]. Meat color was measured in triplicate on the muscle surface after 30 min of blooming at 4 °C, using a calibrated Konica Minolta CR-400 Chroma Meter (Osaka, Japan). Measurements included lightness (L*), redness (a*), yellowness (b*), chroma (C*), and hue angle (h°), according to the CIE Lab* system (Colorimetry, 2nd ed.; CIE Publication No. 15.2; International Commission on Illumination, Vienna, Austria, 1986) [35]. Drip loss was determined using meat samples (4.5 × 4.5 × 2.5 cm; average weight: 54.60 ± 2.15 g), which were weighed, placed on a grid in plastic containers, and stored at 5 °C for 24 h; samples were then blotted and reweighed, and drip loss was expressed as percentage weight loss. Cooking loss was assessed on larger samples (6.0 × 6.0 × 2.5 cm; average weight: 84.22 ± 2.01 g) cooked in a water bath at 80 °C for 1 h, cooled under tap water for 30 min, surface-dried, and reweighed; cooking loss was expressed as percentage weight loss. WBSF was measured on three cylindrical cores (Ø 1.27 cm) obtained from cooked samples, cut parallel to the muscle fibers. A Warner–Bratzler blade mounted on a texture analyzer (Perten TVT 6700, Perten Instruments AB, Hägersten, Sweden) was used to cut the cores perpendicular to the fibers; peak shear was recorded as kgf/cm^2^ (kilogram-force per square centimeter). Texture Profile Analysis (TPA) was also performed using the same instrument. Each sample underwent a double compression cycle with the following settings: test speed 2.0 mm/s; compression 30% of sample height; trigger force 5 g; return speed 2.0 mm/s; and 5 s pause between compressions. The parameters recorded from the force–time curve included: hardness (g), cohesiveness, gumminess (g), springiness (mm), and chewiness (g), in accordance with the TexCal software manual (version 4.0.4.67, Perten Instruments, Milan, Italy). Lipid oxidation was assessed by thiobarbituric acid reactive substances (TBARs) assay, following the method of Tarladgis et al. [36]. Absorbance was measured at 532 nm using an Ultrospec 2100 Pro UV/Visible spectrophotometer (Amersham Pharmacia Biotech, Cambridge, UK). Quantification was based on a standard curve (y = 2 × 10^7^x + 0.0046; R^2^ = 0.9999), covering the concentration range of 1 × 10^−6^ to 1 × 10^−5^ M, equivalent to 0.4–4 mg malondialdehyde (MDA)/kg of meat. MDA recovery was validated by spiking samples with a known volume of 0.2 mM TMP solution. TBARs values were expressed as mg MDA/kg meat [37,38].

The antioxidant activity of meat was evaluated using the DPPH radical scavenging assay, following the method of Brand-Williams et al. [39], with minor modifications. Meat samples (5 g) were homogenized with 50 mL of a solution of methanol/water (80:20, *v*/*v*) containing 0.5% formic acid for 1 min at 7000 rpm and centrifuged at 9000 rpm for 10 min. The extraction with methanol solution was performed in duplicate. After methanol removal, the aqueous extracts were used for the DPPH free radical scavenging assay. Aliquots of 100 μL were mixed with 3.9 mL of 0.06 mM DPPH solution in methanol. After 30 min of incubation in the dark at room temperature, absorbance was measured at 515 nm using a UV–Vis spectrophotometer (SPECTROstarNano, BMG Labtech, Ortenberg, Germany). Results were expressed as mean absorbance values, where lower absorbance corresponds to higher radical scavenging activity.

### 4.8. Statistical Analyses

All data are presented as mean ± standard error of the mean (SEM). Statistical analyses were first performed in CTRL animals (T0 vs. T30) to verify the absence of time-dependent changes. As no significant differences were detected, CTRL animals were considered stable and used as reference groups. Accordingly, paired comparisons were focused on GP5 animals to specifically detect diet-related effects against a homogeneous baseline. For hematological parameters, pooled data from EC and LAC animals were analyzed using a two-tailed paired *t*-test (T0 vs. T30). For biochemical parameters, EC and LAC data were combined, and a two-tailed paired *t*-test was applied to compare T0 vs. T30 values. For milk composition parameters, repeated-measures ANOVA was applied to account for within-subject variability across time points (T0, T10, T20, and T30), followed by Bonferroni post hoc tests. For meat quality traits, EC-CTRL and EC-GP5 animals were compared at T30 using Welch’s *t*-test. Differences were considered statistically significant at *p* < 0.05. All analyses were performed using GraphPad Prism version 10.5 (GraphPad Software, La Jolla, CA, USA).

## 5. Conclusions

This study demonstrates that supplementing sheep diets with GP5 is metabolically safe and induces measurable systemic health, metabolic safety and milk-related adaptations while preserving hematological integrity and without compromising clinical status. GP5 supplementation enhanced hepatic protein status, promoted shifts in nitrogen and energy metabolism, and altered milk composition without adverse effects on meat quality. Notably, the marked reduction in milk lactose represents a distinctive compositional shift; while this observation is intriguing, its nutritional implications remain to be elucidated and warrant confirmation through dedicated studies. Collectively, these findings highlight the functional potential of GP5 as a sustainable dietary strategy in small ruminant production. Future studies should extend these findings by incorporating molecular and epigenetic endpoints, such as rumen VFA profiling, hepatic gene expression, and miRNA regulation, to elucidate the mechanistic basis of the observed adaptations. In addition, longer supplementation periods and larger cohorts will be required to confirm the persistence and reproducibility of the effects across breeds and production systems. These directions will allow linking the identified polyphenols to specific metabolic and gene-level pathways, thereby expanding the translational relevance of GP5 supplementation.

## Figures and Tables

**Table 1:** Total phenolic content (TPC), antioxidant activity (DPPH, FRAP, ABTS), and anthocyanin concentration in grape pomace extract.

Analysis	Mean ± SEM (mg/g)
TPC (mgGAE/g DW)	254.02 ± 20.39
DPPH (mgTE/g DW)	1110.97 ± 13.39
FRAP (mgTE/g DW)	1309.63 ± 64.51
ABTS (mgTE/g DW)	139.55 ± 2.92
Anthocyanin (mg of malvidin 3-O-glucoside equivalent/g)	3.94 ± 0.13

Abbreviations: DW, dry weight; GAE, gallic acid equivalents; TE, Trolox equivalents. SEM, standard error of the mean.

**Table 2:** Analytical composition of the GP5 and control pellets (% as fed basis). Values are expressed on an as-fed basis.

Analytical Component	Abbreviation	CTRL	GP5
Crude protein	CP	15.43	15.24
Ether extract (fat)	EE	2.85	2.9
Crude fiber	CF	5.65	7.02
Lignin	LG	3.58	3.93
ASH	ASH	7.4	7.42
Calcium	Ca	0.82	0.83
Total phosphorus	P	0.44	0.45
Sodium	Na	0.2	0.21
Dry matter	DM	86.78	86.92

**Table 3:** Hematological parameters in GP5 animals (EC + LAC pooled) at T0 and T30 (mean ± SEM). Data are expressed as mean ± SEM. Reference values are derived from National Research Council (2007) [15].

Parameter	Reference Range	T0 (Mean ± SEM)	T30 (Mean ± SEM)	*p*-Value
WBC (×10^9^/L)	4–12	10.0 ± 0.6	7.3 ± 2.7	*p* = 0.71
LYM (×10^9^/L)	2.5–9.0	5.0 ± 1.6	3.4 ± 1.1	*p* = 0.43
MONO (×10^9^/L)	0.0–1.0	1.0 ± 0.2	0.7 ± 0.2	*p* = 0.31
GRAN (×10^9^/L)	1.5–7.5	6.8 ± 0.6	3.2 ± 0.2	*p* = 0.56
HGB (g/dL)	8–16	10.6 ± 1.2	10.7 ± 0.9	*p* = 0.95
HCT (%)	24–49	32.9 ± 3.4	33.3 ± 2.6	*p* = 0.93
RBC (×10^12^/L)	9–15	9.9 ± 1.6	9.3 ± 1.7	*p* = 0.80
MCV (fL)	23–48	35.4 ± 1.0	35.6 ± 0.8	*p* = 0.88
MCH (pg)	8–12	11.3 ± 0.6	11.3 ± 0.6	*p* = 1.00
MCHC (g/dL)	31–34	32.4 ± 0.3	32.7 ± 0.3	*p* = 0.50
RDW (%)	12.0–18.5	18.8 ± 1.4	19.2 ± 0.9	*p* = 0.81
RDWa (fL)	20–43	23.5 ± 1.2	23.7 ± 1.1	*p* = 0.90
PLT (×10^9^/L)	200–850	188 ± 4.5	191 ± 6	*p* = 0.97
MPV (fL)	3.6–6.5	5.6 ± 0.4	5.5 ± 0.3	*p* = 0.85

Abbreviations: WBC, white blood cells; LYM, lymphocytes; MONO, monocytes; GRAN, granulocytes; HGB, hemoglobin; HCT, hematocrit; RBC, red blood cells; MCV, mean corpuscular volume; MCH, mean corpuscular hemoglobin; MCHC, mean corpuscular hemoglobin concentration; RDW, red cell distribution width; RDWa, red cell distribution width, absolute; PLT, platelets; and MPV, mean platelet volume.

**Table 4:** Biochemical parameters in GP5 animals (EC + LAC pooled) at T0 and T30 (mean ± SEM). Parameters are grouped by functional category. Data are expressed as mean ± SEM. Exact *p*-values for paired comparisons between T0 and T30 are reported in the last column; values < 0.05 are considered statistically significant (two-tailed paired *t*-test). Superscript letters (a, b) indicate significant differences between time points. Most parameters remained within or close to physiological reference ranges; bilirubin at T30 slightly exceeded the upper limit, without clinical signs. Reference ranges are laboratory-specific (see Section 4.5) Reference values are derived from National Research Council (2007) [15].

Metabolic Profile	Parameter	Reference Range	T0 (Mean ± SEM)	T30 (Mean ± SEM)	*p*-Value
Hepatic and biliary enzymes	ALB	21–36 g/L	33.21 ± 2.63 ^a^	37.76 ± 4.48 ^b^	*p* = 0.0181
	ALP	68–387 U/L	140.64 ± 97.55	83.09 ± 37.85	*p* = 0.1891
	ALT	22–38 U/L	16.55 ± 5.03	21.82 ± 9.06	*p* = 0.1140
	AST	60–280 U/L	129.55 ± 64.32	103.09 ± 33.83	*p* = 0.3408
	GGT	20–52 U/L	49.18 ± 14.65	55.64 ± 5.64	*p* = 0.1483
	BIL	1.7–8.5 µmol/L	7.25 ± 4.42 ^a^	10.76 ± 5.99 ^b^	*p* = 0.0302
Renal Function	UREA	2.8–7.1 mmol/L	9.56 ± 1.48 ^a^	6.33 ± 1.12 ^b^	*p* < 0.05
	CREA	106–168 µmol/L	52.54 ± 20.18 ^a^	70.97 ± 13.26 ^b^	*p* = 0.0086
Metabolic Indicators—Muscle Enzymes	CPK	8.1–12.9 U/L	141.27 ± 81.38	164.55 ± 64.20	*p* = 0.2372
	CHOL	1.35–1.97 mmol/L	1.57 ± 0.29	1.61 ± 0.29	*p* = 0.8880
	TG	0.1–0.5 mmol/L	0.19 ± 0.10 ^a^	0.32 ± 0.10 ^b^	*p* = 0.0164
	TP	60–80 g/L	74.32 ± 8.69	75.67 ± 10.40	*p* = 0.5545
	KET	0.5–0.9 mmol/L	0.21 ± 0.12	0.32 ± 0.25	*p* = 0.4701
	GLU	2.8–4.4 mmol/L	3.19 ± 0.22 ^a^	2.22 ± 0.13 ^b^	*p* < 0.05
	LDH	800–1300 U/L	1020.18 ± 367.37	1209.73 ± 209.88	*p* = 0.2122
Mineral Metabolism	Ca	2.6–3.2 mmol/L	2.32 ± 0.37	2.50 ± 0.20	*p* = 0.3409
	P	1.6–2.4 mmol/L	1.70 ± 0.74 ^a^	1.55 ± 0.35 ^a^	*p* = 0.9215

Abbreviations: ALB, albumin; ALP, alkaline phosphatase; ALT, alanine aminotransferase; AST, aspartate aminotransferase; GGT, gamma-glutamyl transferase; BIL, bilirubin; UREA, urea; CREA, creatinine; CPK, creatine phosphokinase; CHOL, cholesterol; TG, triglycerides; TP, total protein; KET, ketone bodies; GLU, glucose; LDH, lactate dehydrogenase; Ca, calcium; P, phosphorus.

**Table 5:** Milk composition parameters in the GP5 group at T0, T10, T20, and T30. Data are expressed as mean ± SEM.

Parameter	T0 (Mean ± SEM)	T10 (Mean ± SEM)	T20 (Mean ± SEM)	T30 (Mean ± SEM)	Overall *p*-Value
Lactose (%)	4.66 ± 0.09 ^a^	2.95 ± 0.26 ^b^	1.71 ± 0.23 ^c^	0.87 ± 0.42 ^c^	*p* < 0.05
Urea (mg/dL)	58.31 ± 2.43 ^a^	50.48 ± 2.40 ^a^	47.57 ± 3.98 ^a^	17.26 ± 1.91 ^b^	*p* < 0.05
pH	6.80 ± 0.08 ^a^	6.80 ± 0.08 ^a^	7.31 ± 0.06 ^b^	7.39 ± 0.05 ^c^	*p* < 0.05
Acidity (°SH)	5.40 ± 0.50 ^a^	5.18 ± 0.48 ^a^	4.40 ± 0.48 ^a^	3.68 ± 0.17 ^b^	*p* < 0.05
Fat (%)	6.79 ± 0.33 ^a^	6.83 ± 0.54 ^a^	6.47 ± 0.53 ^a^	5.02 ± 0.61 ^a^	*p* = 0.1416
Caseins (%)	4.32 ± 0.12 ^a^	4.28 ± 0.17 ^a^	3.96 ± 0.16 ^a^	3.87 ± 0.26 ^a^	*p* = 0.0937
Proteins (%)	6.40 ± 0.58 ^a^	6.42 ± 0.16 ^a^	6.62 ± 0.35 ^a^	6.09 ± 0.44 ^a^	*p* = 0.8843
Freezing point (°C)	−0.54 ± 0.00 ^a^	−0.55 ± 0.01 ^a^	−0.53 ± 0.01 ^a^	−0.53 ± 0.00 ^a^	*p* = 0.0720

Different superscript letters within a row indicate significant post hoc differences (*p* < 0.05).

**Table 6:** Physicochemical, texture, and oxidative parameters of Longissimus dorsi muscle in EC animals at T30. Values are expressed as mean ± SEM. Exact *p*-values are reported in the last column; values < 0.05 are considered statistically significant.

Parameter	EC-CTRL T30 (Mean ± SEM)	EC-GP5 T30 (Mean ± SEM)	*p*-Value
pH	5.95 ± 0.05	5.95 ± 0.20	*p* = 0.99
L* (Lightness)	33.22 ± 0.98	35.66 ± 3.41	*p* = 0.54
a* (Redness)	18.89 ± 0.87	17.64 ± 0.88	*p* = 0.35
b* (Yellowness)	6.49 ± 0.53	6.65 ± 0.29	*p* = 0.80
Drip loss (%)	1.71 ± 0.17	1.43 ± 0.20	*p* = 0.34
Cooking loss (%)	35.71 ± 0.94	37.97 ± 2.21	*p* = 0.50
WBSF (kgf/cm^2^)	8.81 ± 0.30	10.10 ± 0.53	*p* = 0.36
Hardness (g)	1418.00 ± 222.82	883.00 ± 134.83	*p* = 0.10
Springiness	0.56 ± 0.02	0.61 ± 0.05	*p* = 0.41
Chewiness (g)	481.20 ± 89.93	273.00 ± 46.27	*p* = 0.10
Gumminess (g)	858.00 ± 178.20	456.25 ± 84.01	*p* = 0.08
Cohesiveness	0.60 ± 0.06	0.52 ± 0.05	*p* = 0.07
TBAR (mg MDA/kg)	0.35 ± 0.03	0.37 ± 0.04	*p* = 0.66
DPPH (Abs 515 nm)	0.48 ± 0.11	0.34 ± 0.04	*p* = 0.12

**Table 7:** Composition of raw ingredients included in the CTRL and GP5 pellets used in the nutritional trial. The only modification between the diets was the inclusion of 5% grape pomace, compensated by small reductions in wheat by-products (bran −2.0; middlings −2.2, % as-fed). The detailed composition of the mineral–vitamin premix (0.30%) is provided in Supplementary Table S2.

Ingredient (%)	CTRL	GP5
Maize	27.80	27.00
Durum wheat bran	27.00	25.00
Durum wheat middlings	26.50	24.30
Soybean meal (48% CP)	5.30	5.30
Defatted maize germ meal	5.00	5.00
Molasses solubles	2.50	2.50
Calcium carbonate	2.00	2.00
Cane molasses	2.00	2.00
Sodium bicarbonate	0.80	0.80
Sodium chloride	0.50	0.50
Urea (46% N)	0.30	0.30
Mineral–vitamin premix	0.30	0.30
Grape pomace	0.00	5.00

## Data Availability

The original contributions presented in this study are included in the article/Supplementary Materials. Further inquiries can be directed to the corresponding authors.

## Supplementary Materials

The following supporting information can be downloaded at: https://www.mdpi.com/article/10.3390/ijms262110578/s1.
