# Supplementary material for: Sustainable Valorization of Grape Pomace in Sheep Through Systemic Health, Metabolic Safety, Milk and Meat Quality
Source: Int J Mol Sci. 2025 Oct 30;26(21):10578. doi: 10.3390/ijms262110578 (PMC12609406; doi:10.3390/ijms262110578)
Supplement: Supplementary file 1 [file ijms-26-10578-s001.zip › ijms-3930393-Supplementary.pdf]

Supplementary Table S1.

Identified compounds in grape pomace extract by UHPLC–HRMS. Peaks correspond to those detected in the base peak chromatogram (Figure S2), with molecular formula, exact mass, and fragmentation profile. The notation nf indicates cases where no characteristic MS/MS fragments were detected or fragmentation data were not available.

Pk. no.	RT (min)	$[M-H]^-$ *m/z* Calculated	$[M-H]^-$ *m/z* Observed	Predicted Molecular Formula	MS/MS *m/z*	Compound Identity
**1**	1.81	191.0561	191.0568	$C_7H_{12}O_6$	85 (100)	Quinic acid
**2**	1.92	133.0143	133.0144	$C_4H_6O_5$	115 (100)	Malic acid
**3**	1.95	149.0081	149.0092	$C_4H_6O_6$	103, 87 (100), 72, 59	Tartaric acid
**4**	3.21	117.0182	117.0194	$C_4H_6O_4$	99, 73 (100)	
**5**	8.09	331.066	331.067	$C_{13}H_{16}O_{10}$	169 (100)	
**6**	8.86	577.1352	577.1351	$C_{30}H_{26}O_{12}$	407, 289 (100), 245, 203, 201, 161, 125	Procyanidin B2
**7**	9.63	289.0718	289.0716	$C_{15}H_{14}O_6$	245, 203, 151, 125, 123, 109 (100)	+/- Catechin
**8**	9.96	577.1352	577.1351	$C_{30}H_{26}O_{12}$	407, 289 (100), 245, 203, 201, 161, 125	Procyanidin like
**9**	10.52	729.145	729.1459	$C_{30}H_{34}O_{21}$	407 (100), 289, 169, 125	
**10**	10.9	289.0718	289.0716	$C_{15}H_{14}O_6$	245, 203, 151, 125, 123, 109 (100)	Epicatechin
**11**	11.83	197.0445	197.0455	$C_9H_{10}O_5$	nf	
**12**	12.09	441.0816	441.0826	$C_{22}H_{18}O_{10}$	203 (100), 201, 169, 125	
**13**	12.48	577.1352	577.1355	$C_{30}H_{26}O_{12}$	407, 289 (100), 245, 203, 201, 161, 125	Procyanidin like
**14**	12.72	509.129	509.1302	$C_{23}H_{26}O_{13}$	nf	
**15**	13.73	363.0711	363.072	$C_{17}H_{16}O_9$	nf	
**16**	14.66	655.1658	655.1669	$C_{25}H_{36}O_{20}$	nf	
**17**	14.66	463.0882	463.0882	$C_{21}H_{21}O_{12}$	300 (100)	Quercetin 3-*β*-d-glucoside
**18**	15.7	477.0664	477.0675	$C_{21}H_{18}O_{13}$	nf	
**19**	16.32	271.0612	271.0611	$C_{15}H_{12}O_5$	151 (100)	Naringenin
**20**	17.36	301.0354	301.0352	$C_{15}H_{10}O_7$	203, 178, 151 (100), 121	Quercetin
**21**	18.58	613.343	613.3442	$C_{28}H_{54}O_{14}$	493 (100), 475, 331, 2023	
**22**	18.88	285.0405	285.0404	$C_{15}H_9O_6$	203, 151 (100), 107	Luteolin
**23**	20.54	207.138	207.139	$C_{13}H_{20}O_2$	152, 135, 133, 121, 101 (100), 55	
**24**	21.37	313.0707	313.0717	$C_{17}H_{14}O_6$	225, 203, 181, 121 (100)	
**25**	23.48	383.0761	383.0769	$C_{20}H_{16}O_8$	339, 311, 225, 203, 191, 121 (100)	
**26**	26.38	471.3479	471.348	$C_{30}H_{48}O_4$	203 (100)	Maslinic acid
**27**	27.97	367.2632	367.2639	$C_{25}H_{36}O_2$	273, 225, 203, 177 (100)	
**28**	28.06	455.3531	455.3529	$C_{30}H_{47}O_3$	203 (100)	Ursolic acid
**29**	28.21	279.2319	279.2328	$C_{18}H_{32}O_2$	261, 225, 203 (100), 176, 148	

Supplementary Figure S1

Base peak chromatogram of grape pomace extract analyzed by UHPLC–HRMS in negative ionization mode. Peaks 1–29 correspond to the polyphenolic compounds identified and listed in Table S1.

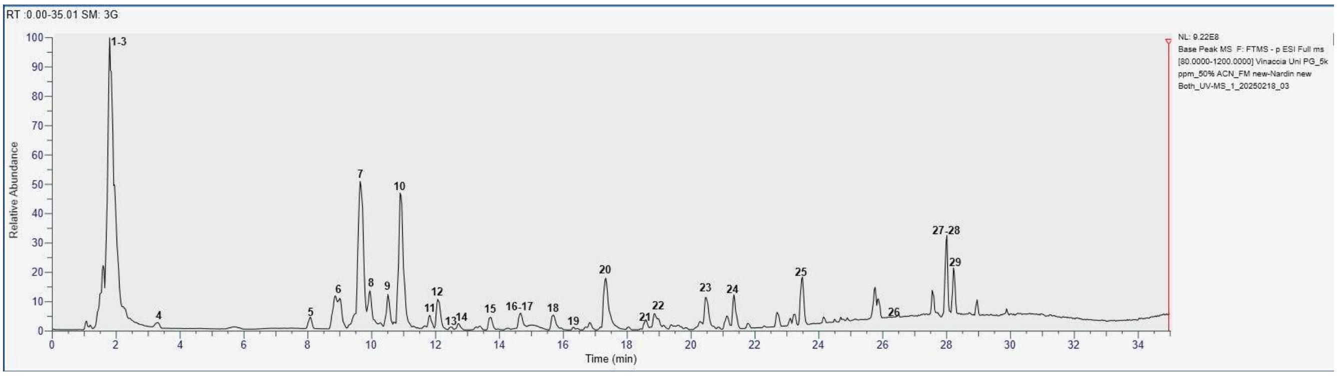

Supplementary Table S2

Composition of the mineral–vitamin premix for dairy sheep (per kg of premix).

**Additive**	**Content per kg premix**
**Vitamins**	
Vitamin A (3a672a, retinyl acetate)	8,000,000 IU
Vitamin D_3 (3a671, cholecalciferol)	600,000 IU
Vitamin E (3a700i, all-rac-α-tocopheryl acetate)	10,000 mg
Vitamin B_1 (3a821, thiamine mononitrate)	400 mg
Vitamin B_2 (3a825i, riboflavin)	200 mg
Calcium D-pantothenate (3a841)	1,000 mg
Vitamin B_6 (3a831, pyridoxine HCl)	200 mg
Vitamin B_{12} (3a835, cyanocobalamin)	4.0 mg
Nicotinic acid (3a314)	20,000 mg
Biotin (3a880)	10.0 mg
Choline chloride (3a890)	20,000 mg
**Trace elements**	
Calcium iodate anhydrous, coated (3b203, I)	600 mg
Manganese oxide (3b502, Mn)	10,000 mg
Sodium selenite, coated (3b802, Se)	60 mg
Zinc oxide (3b603, Zn)	20,000 mg
**Carrier**	Calcium carbonate, grape pomace, calcium and magnesium carbonate (q.s. to 1000 g)

Supplementary Table S3

Milk composition parameters in the CTRL group at T0, T10, T20, and T30, and in the GP5 group at T0. Data are expressed as mean ± SE. One way ANOVA followed by Bonferroni post-hoc tests was used to evaluate statistical differences. No statistically significant differences have been detected between groups and time points ($p > 0.05$). P-values are reported in the last column. Somatic cell count (SCC) values are also reported; physiological thresholds for ovine milk are < 500 × 10^3 cells/mL.

Parameter	T0 CTRL (mean ± SE)	T10 CTRL (mean ± SE)	T20 CTRL (mean ± SE)	T30 CTRL (mean ± SE)	T0 GP5 (mean ± SE)	p-value
**Lactose (%)**	4.40 ± 0.18	4.25 ± 0.17	4.00 ± 0.30	3.98 ± 0.28	4.66 ± 0.09	p > 0.05
**Urea (mg/dL)**	60.75 ± 3.77	61.48 ± 4.36	61.45 ± 3.00	53.70 ± 3.36	58.31 ± 2.43	p > 0.05
**pH**	6.78 ± 0.02	6.80 ± 0.04	6.76 ± 0.05	6.58 ± 0.03	6.80 ± 0.08	p > 0.05
**Acidity (°SH)**	5.86 ± 0.66	5.82 ± 0.73	7.18± 0.43	7.20 ± 0.55	5.40 ± 0.50	p > 0.05
**Fat (%)**	6.44 ± 0.48	8.90 ± 0.51	6.35 ± 0.62	7.44 ± 0.38	6.79 ± 0.33	p > 0.05
**Caseins (%)**	4.41 ± 0.22	4.54 ± 0.20	4.06 ± 0.30	4.42 ± 0.15	4.32 ± 0.12	p > 0.05
**Proteins (%)**	5.54 ± 0.20	5.92 ± 0.38	6.01 ± 0.31	5.90 ± 0.25	6.40 ± 0.58	p > 0.05
**Freezing point (°C)**	-0.53 ± 0.01	-0.54 ± 0.00	-0.54 ± 0.00	-0.54 ± 0.00	-0.54 ± 0.00	p > 0.05
**SCC (×10^3 cells/mL)**	< 500	< 500	< 500	< 500	< 500	-
